# Shortcomings in the Cochrane review on zinc for the common cold (2024)

**DOI:** 10.3389/fmed.2024.1470004

**Published:** 2024-10-16

**Authors:** Harri Hemilä, Elizabeth Chalker

**Affiliations:** ^1^Department of Public Health, University of Helsinki, Helsinki, Finland; ^2^National Centre for Epidemiology and Population Health, Australian National University, Canberra, ACT, Australia

**Keywords:** adverse effects, common cold, meta-analysis, pharmacology, randomized controlled trials, respiratory tract infections, zinc acetate, zinc lozenges

## Introduction

Interest in zinc lozenges (tablets to be dissolved slowly in the mouth) for common cold treatment started from the serendipitous observation that the cold symptoms of a 3-year-old girl with leukemia disappeared within a few hours when she allowed a zinc tablet to slowly dissolve in her mouth instead of swallowing it whole (1, 2). The benefit appeared to be derived from dissolving (rather than swallowing) the tablet, which implied that zinc may have local effects in the oropharyngeal region. This observation led the father of the child, George Eby, to conduct a randomized controlled trial (RCT), which found that zinc gluconate lozenges significantly shortened colds (1), and increased the recovery rate from the common cold with a rate ratio (RR) of 3.5 (95% CI: 1.8–6.7) compared to placebo (3).

Subsequently, over a dozen placebo-controlled trials were carried out with varying results, with the composition of the lozenges and the dose of zinc effectively explaining the variation (2, 4). In seven RCTs, zinc acetate and zinc gluconate lozenges containing >75 mg/day of elemental zinc shortened common cold duration on average by 33% (95% CI: 21%−45%, *P* = 10^−7^) (5). In three zinc acetate lozenge trials, the rate of recovery from the common cold increased with a RR of 3.1 (95% CI: 2.1–4.7) (3). In these zinc acetate trials, there was no substantial difference between the size of the effect on nasal symptoms, sore throat and cough (6), or by age, gender, ethnic group, allergy status, smoking, or baseline cold severity (7). Thus, there is strong evidence that appropriately composed zinc lozenges, especially zinc acetate lozenges, can help to treat colds.

In 2011, a Cochrane review on zinc for the common cold was published (8). Several errors were pointed out and revisions were suggested (9), however, the 2013 update contained essentially the same errors. There was an additional concern of plagiarism which led to the withdrawal of the review in 2015 (10, 11) and retraction of the associated JAMA summary (12–14).

In 2024, a new Cochrane review was published on zinc for the common cold (15). The review concluded that “*On the basis of this review, the current evidence is insufficient to provide firm conclusions or recommend zinc supplementation for the prevention or treatment of the common cold*” [(15), p. 28]. This conclusion is quite different to the conclusions in the meta-analyses described above (2–7). Therefore, we critically read the new Cochrane review and describe here several concerns which explain the different conclusions, with a detailed description elsewhere (16).

## Cochrane review on zinc for the common cold (2024)

### Revision of the main meta-analysis on common cold treatment

The Cochrane review's conclusion on zinc treatment for the common cold is based on Analysis 9.1 [(15), p. 2 and 163]: “*When zinc is used for cold treatment, there may be a reduction in the mean duration of the cold in days (MD* −*2.37, 95% CI* −*4.21 to* −*0.53; I*^2^ = *97%; eight studies)*.” This Cochrane estimate has a very wide confidence interval, indicating that the efficacy appears doubtful. However, the calculation is not appropriate.

First, mean difference (MD) is a measure of average treatment effect in days, but this is a poor measure for the effect of common cold treatments. Colds can last for 1 day or 3 weeks and over. Obviously, 1-day colds cannot be shortened by 2.37 days. It has been shown that the relative scale (percentage effect) much better captures the treatment effect on the duration of illness (17–22). A scale which better fits the data also leads to more accurate estimates and narrower confidence intervals. We used the relative scale in our Cochrane review on vitamin C for the common cold (23).

Second, the authors write in the Methods section that “*We undertook meta-analyses only where meaningful, that is, if the treatments, participants, and the underlying clinical question(s) were similar enough for pooling to make sense”* [(15), p. 10]. However, Analysis 9.1 includes both zinc lozenge trials and nasal zinc administration trials, although they are not similar treatments. Furthermore, one of the included zinc lozenge trials administered 190 mg/day zinc (24) whereas one nasal zinc trial administered just 0.046 mg/day (25). Pooling two trials with a 4,300-fold difference in the dose is not meaningful.

Third, the Cochrane authors do not justify their exclusion of the Mossad et al. (26) trial from Analysis 9.1, even though it was a placebo-controlled RCT. The Cochrane authors include this trial in several other comparisons (15), indicating that they had no concerns with the methodology.

Fourth, one of the zinc lozenge trials included in the calculation of the 2.37-day estimate was carried out with children (27). There can be differences in the size of the effect between adults and children and therefore we separated adults and children in our Cochrane review on vitamin C for the common cold (23).

**Figure 1:**
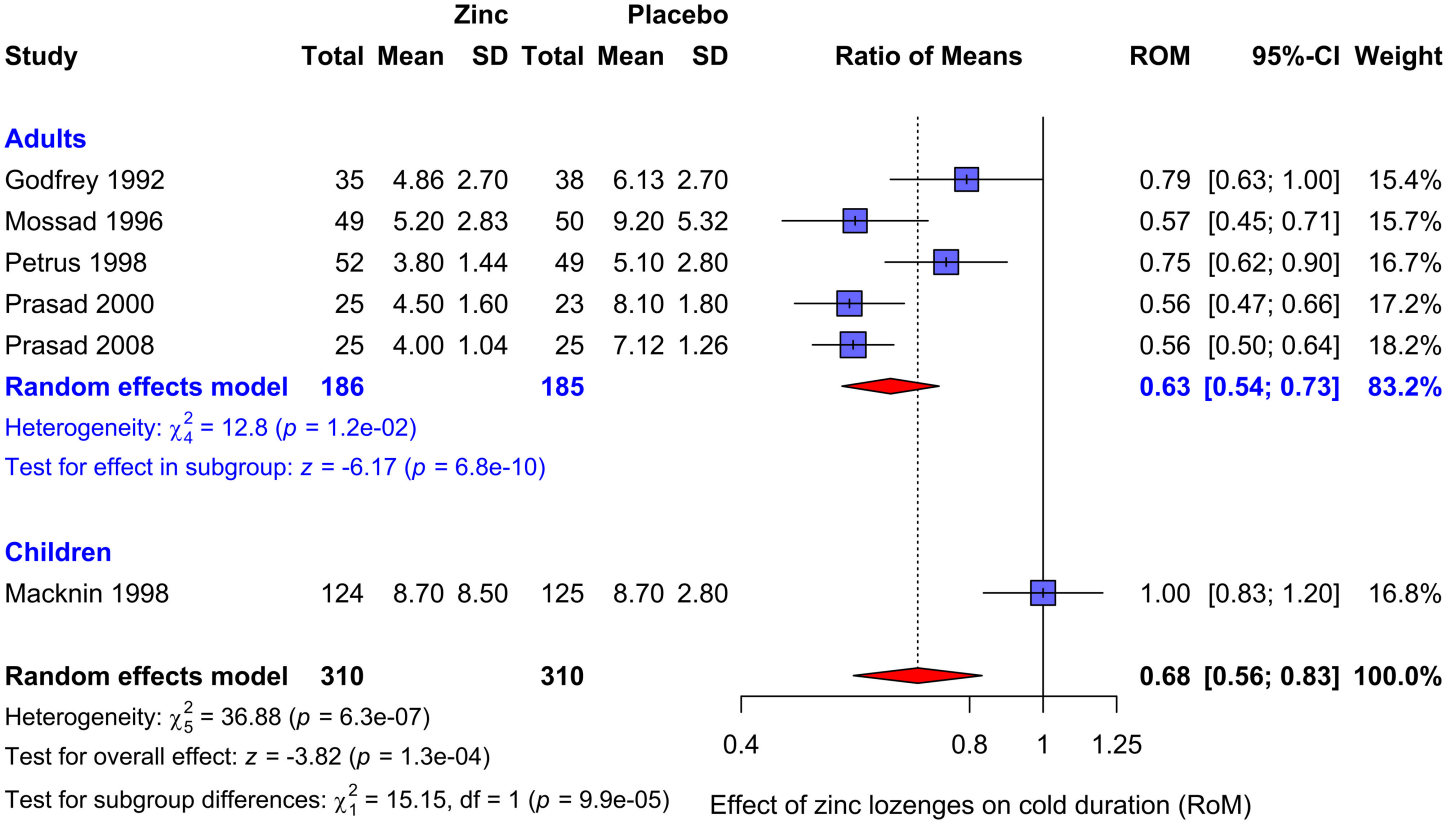
Pooling the zinc lozenge trials included in the Cochrane review on zinc for the common cold (2024) (15). Zinc lozenges shortened colds in adults with a RoM of 0.63, that is, by 37% (95% CI: 27%−46%; *P* = 10^−9^). The child trial by Macknin et al. (27) is inconsistent with the trials with adults (*P* = 0.0001 for the test of heterogeneity) and should be kept separated. The data are from Nault et al. (15), except for the Mossad et al. (26) trial, which are from Hemilä (19). We pooled the included trials with the *metagen* function of the R package *meta*, using the inverse variance, random effects options (28). RoM, ratio of means (18); RoM = 1.0 indicates that the mean duration of colds is identical in the intervention and control groups.

We revised the meta-analysis corresponding to Analysis 9.1 (15) by restricting the analysis to zinc lozenges, including the Mossad et al. (26) trial, and using the relative scale, and calculated that zinc lozenges shortened colds in adults by 37% (95% CI: 27%−46%; *P* = 10^−9^), see Figure 1. This provides very strong evidence that zinc lozenges can shorten common colds in adults, consistent with the previous analyses (2–7).

Inclusion of the trial with children (27) has a minimal effect on the pooled estimate (Figure 1). However, there is a highly significant difference between the pooled estimate for the five adult trials and the trial with children (*P* = 0.0001). Therefore, it is most informative to keep the adult and child trials separated. The published trials indicate that appropriately composed zinc lozenges can shorten colds in adults by about 37%, but so far there is no evidence that zinc lozenges shorten colds in children.

### Pharmacology of zinc lozenges is not considered

The Cochrane authors consider that zinc lozenges and nasal zinc are forms of dietary supplementation: “*Zinc is naturally present in some foods (e.g. red meat), is sometimes added to other foods (e.g. zinc fortified cereals), and may be taken as an over-the-counter dietary supplement… Zinc supplements exist in several forms, including zinc gluconate, zinc sulfate, zinc acetate, zinc carnosine, and zinc picolinate, which vary in percentage of elemental zinc… Zinc is a popular supplement often recommended to reduce the duration of the common cold*” [(15), p. 8].

According to the US Food and Drug Administration, “*A dietary supplement is a product intended for ingestion that, among other requirements, contains a “dietary ingredient” intended to supplement the diet”* (29).

The Cochrane review does not consider the pharmacology of zinc lozenges. In particular, zinc administered nasally is not ingested. Mossad (30) administered 2.1 mg/day zinc nasally in a gel and colds were shortened by 37% (95% CI: 22%−49%). The benefit of nasal zinc is definitively inconsistent with the concept that the mode of action is through dietary supplementation.

If the effects of zinc lozenges and nasal zinc are local as proposed by Eby (1, 2), optimal formulation of the lozenges is essential. Some of the zinc lozenges examined in the trials contained citric acid, tartaric acid, mannitol-sorbitol, or other substances that bind zinc so that it is not freely released in the oropharyngeal region (2, 16, 31, 32). Eby showed close correlation between the calculated free zinc dose and the efficacy in RCTs, consistent with the importance of lozenge composition (2). This also means that if the goal is to estimate the efficacy of optimally composed zinc lozenges, a meta-analysis should include only lozenges that release zinc effectively. This issue was not considered in the Cochrane review (15).

### Discussion of adverse effects is not appropriate

The Cochrane review concludes in the abstract (15): “*There is probably an increase in the risk of non-serious adverse events when zinc is used for cold treatment (RR 1.34 …).”* This analysis has two shortcomings. First, the variation in lozenge composition is not taken into account. Second, estimating the size of the adverse effects as a RR is misleading when the focus is on mild adverse effects.

The most usual minor adverse effects relate to taste. In his review, Eby pointed out that the taste problems of zinc lozenges largely depend on the composition of the lozenges: “*Due to serious taste issues zinc gluconate was a poor choice for treating colds. Zinc gluconate forms extremely bitter complexes with all sweet carbohydrates except fructose… The overriding source of failure was requirement by pharmaceutical marketing companies for pleasant tasting, candy-like, non-metallic, non-astringent and non-drying zinc lozenges”* [(2), p. 488]. However, the composition of the lozenges is not considered in the Cochrane review (15).

The RR between the placebo and treatment groups is a meaningful measure for rare severe adverse events, but not for minor adverse effects. All examined zinc lozenges caused taste and mouth effects for some patients: sour taste, sweet taste, bad taste, mouth dryness and mouth irritation (2, 16). This means that an ideal placebo lozenge would cause similar reactions. Thus, if an ideal placebo lozenge was compared with the respective zinc lozenge, it's likely there would be no difference in the rate of minor adverse effects with RR = 1.0. However, that does not mean that the adverse effects of the particular zinc lozenge are null. Both placebo and zinc lozenges could taste so awful that no patient will continue usage, but that would not be captured by the calculation of the RR. Instead, more useful measures to assess minor adverse effects of zinc lozenges are to consider the proportion of patients who do not complain of adverse effects from the zinc lozenges, and the proportion who continue until the trial ends. The mild adverse effects of properly composed zinc lozenges have not been so severe that patients were unwillingly to continue treatment (16). If a patient considers that the taste is too unpleasant, they can cease treatment any time.

As to severe adverse effects, high doses of zinc have been given to patients with various diseases for several months without concerns (3–7, 16). Furthermore, zinc is a standard treatment for Wilson's disease, which usually means taking high doses long-term (16, 33–35). In the treatment of Wilson's disease, 150 mg/day of zinc has had an excellent safety profile, though it has caused gastric irritation in 5–10% of patients (35). Thus, it seems highly unlikely that 80–92 mg/day of zinc in the zinc lozenge trials (2–7), for 1–2 weeks would cause long-term severe adverse effects.

## Discussion

The common cold is the leading cause of acute morbidity and visits to physicians in high-income countries, and it is a major cause of absenteeism from work and school. In one analysis, the economic burden of the common cold was comparable to hypertension and stroke (36).

Using antibiotics to treat a typical acute common cold episode is useless because most colds are caused by viruses. Nevertheless, according to some surveys, about half the common cold patients in the USA received antibiotics (37, 38). In this respect, alternative treatment options for the common cold have substantial public health relevance (23, 39–42), and the possibility of shortening colds with zinc acetate lozenges is important (2–7). Unfortunately, the conclusions of the Cochrane review on zinc for the common cold (2024) (15) are based on flawed statistical analyses.

Although properly composed zinc lozenges can shorten the duration of colds in adults by 30%−40% [Figure 1 and (4–7)], it is not easy for common cold patients to find effective lozenges (2, 43). Eby wrote in his 2010 review that “*Zinc lozenges marketed in the United States appear to compete based upon taste rather than efficacy… Of the 40 different brands of over-the-counter zinc lozenges and many variations of them currently available in the US, very few—based upon this analysis and ingredients listed on their labels—appear to release useful amounts of iZn [free zinc ions] regardless of total zinc content, and none of them can be considered as a cure for common colds. With several exceptions, nearly all appear likely to have a null effect on colds.”* [(2), p. 490]. However, this can be overcome with advice from health practitioners to seek lozenges that have appropriate levels of zinc and do not contain citric acid.
